# Prevalence of Ovine Haemonchosis in Wukro, Ethiopia

**DOI:** 10.1155/2015/635703

**Published:** 2015-01-22

**Authors:** Lidya Gebresilassie, Berihun Afera Tadele

**Affiliations:** ^1^Tigray Bureau of Agriculture and Rural Development Office, Ethiopia; ^2^College of Veterinary Medicine, Mekelle University, P.O. Box 231, Mekelle, Ethiopia

## Abstract

*Background*. *Haemonchosis* caused by *Haemonchus contortus* is a predominant, highly pathogenic, and economically important disease of sheep and goats. *Objective*. Assessing the prevalence of *Haemonchus* parasite and its associated risk factors in sheep slaughtered at different restaurants of Wukro. *Methods*. Cross-sectional study using random sampling from November 2013 to April 2014 in a total of 384 sheep was conducted and SPSS version 20 software using descriptive statistics was used for data analysis and *P* < 0.05 was considered significant. *Result*. The overall prevalence of *Haemonchus contortus* was 40.9% (*n* = 157). The prevalence in medium body condition 27.3% (*n* = 105) varies significantly from that of good body condition 13.5% (*n* = 52) (*P* < 0.05). Moreover, there was significant variation (*P* < 0.05) in the prevalence in young and adult sheep with rates of 21.9% (*n* = 84) and 19% (*n* = 73), respectively. At the same time, there is significant variation (*P* < 0.05) in male and female sheep with prevalence of 29.7% (*n* = 114) and 11.2% (*n* = 43), respectively. The prevalence of 25.3% (*n* = 97) in sheep that originated from Negash compared to Wukro and Agulae showed no significant variation (*P* > 0.05). *Conclusion*. The current finding revealed that significant numbers of sheep were affected by the parasites. Hence strategic deworming with good husbandry practice should be implemented.

## 1. Background

In many countries particularly, small ruminants play a great role in the economy of the country, as sources of meat, milk, fiber, cash income, and skin and they can live in extreme climatic conditions, they can exploit herbage, which is unsuitable for large ruminants, and they require few labor-intensive inputs [1].

Ethiopia lies within the tropical latitudes of Africa and has an extremely diverse topography, wide range of climatic features, and multitude of agroecological zones, which make the country suitable for different agricultural production systems. This in turn has contributed to the existence of a large diversity of farm-animal genetic resources in the country. Ethiopian livestock production systems are broadly characterized as low input, mixed crop-livestock, agropastoral, and pastoral systems, as well as medium input, periurban, and urban enterprises. These livestock are almost entirely managed by the poor smallholder farmers and pastoralists [2].

Ethiopia has the largest livestock and draft animal population in the African continent which is estimated to around 30–40 million tropical livestock units (TLU) out of the total population in Africa. There are approximately 49,297,898 cattle, 25,017,218 sheep, 21,884,222 goat, 7,582,625 equine, 759,696 camels and 38,127,504 chickens are found in the country [3].

Generally, sheep are the predominant livestock in area of high lands of 3,500 meters above sea level; sheep assume a great share in socio-economic activities of about 85% of the population [4].

Small holders in the high land area where is mixed crop-livestock population is practiced own most sheep in Ethiopia, these sheep are an integral part of the livestock sector of the economy. Sheep supply meat, wool/hair and skin that generate about 89% of the farmer's cash income [5].

Other special attributes of sheep over the other livestock resources include that they are highly adaptable to broad ranges of environment, have short generation cycles, and have high reproductive rates which lead to high production efficiency and poor people can afford few ewes since cost of them is less than a cow. With little inputs, sheep play an important role in the rural economy through provision of meat, milk, cash income, accumulating capital, fulfilling cultural obligations, manure and contribute to the national economy which can be incurred due to the export of live animals, meat, and skins [6].

Despite the large livestock population of Ethiopia, the economic benefits remain marginal due to prevailing diseases, poor nutrition, and poor animal production system, reproductive in efficiency, management constraints, and general lack of veterinary care. These diseases have a major impact on morbidity and mortality rates, with annual losses as high as 30–50% of the total value of livestock products of Ethiopia [6]. Endoparasites are responsible for the death of one-third of calves, lambs, and kids and considerable losses of parts of carcasses condemned during meat inspection. It is well recognized that, in resource poor regions of the world, helminthes infections of sheep and goats are major factors responsible for economic losses through reduction in productivity and increased mortality [7].

Nematode parasites of small ruminants result in low productivity due to stunted growth, poor weight gain, and poor feed utilization [8].

In the tropics, the most important nematode species affecting small ruminants are* Haemonchus contortus*,* Trichostrongylus* species,* Cooperia* species,* Bunostomum* species, and* Oesophagostomum* species, whereas* Nematodirus* species are found in the high land areas of most of the country [9, 10].

The principal abomasal worms of sheep are* Haemonchus contortus*,* Ostertagia circumcincta*,* Ostertagia trifurcata*, and* Trichostrongylus axei*.* Haemonchus contortus* is one of the most important abomasal worms of sheep which is known as “red stomach worm” or “wire worm” of small ruminants. It is most prevalent and pathogenic parasite and also economically important disease of sheep [11].* Haemonchus contortus* is a species most commonly found in sheep and goat but* Haemonchus placei* is the usual species in cattle and even so cross infection may occur when small ruminants and cattle graze together but the infestations are usually of less severity [12].

Although helminthes parasites of ruminant livestock are ubiquitous in all of the agroclimatic zones of Ethiopia with prevailing weather conditions that provide favorable condition for their survival and development, their presence does not mean that they cause overt disease. Among the diseases that constrain the survival and productivity of sheep, gastrointestinal nematode infection ranks highest on a global index with* Haemonchus contortus* being of overwhelming importance [13].

*Haemonchus contortus* found in abomasum of sheep and goat causes blood loss resulting in decrease in erythrocytes, lymphocytes, hemoglobin, packed cell volume, body weight, and wool growth. The abomasal nematode* Haemonchus contortus,* which is particularly important and causes severe anemia and death in severely infected animals, identified haemonchosis as one of the top ten constrains to sheep and goat rearing in east Africa [13].

*Haemonchus* is one of the important endoparasites of sheep. The first and second stages of larvae are free-living organisms and the host ingests the third stage larvae starting the infection. Adults of the parasite are found on the surface of the mucosa (the lining of the stomach). Both the larvae (L4) and the adults of* Haemonchus* species suck blood. A thousand* Haemonchus* species of adult can suck 50 mL of blood/day causing severe anemia. A heavy* Haemonchus* species infection (20,000–30,000 worms) can kill sheep very quickly. All ages of sheep are susceptible to* Haemonchus* species infection but lambs are more susceptible than adults [14].

The cardinal sign of haemonchosis is pallor of the skin and mucous membranes. A hematocrit reading of less than 15% is always accompanied by extreme weakness and shortness of breath and warrants a grave prognosis; less of plasma protein results in anasarca frequently manifested externally as a submaxillary edema (bottle jaw). The appetite typically remains good and, in acute outbreaks, affected animals may not lose appreciable weight. Feces are well formed, diarrhea occurring only in infections complicated by the presence of such species as* Trichostrongylus* species and* Cooperia* species. Lambs are the most seriously affected members of the flock, but older sheep under stress also may have fatal anemia [15].

While, in temperate regions, the severity of gastrointestinal (GI) parasitic disease in most livestock farms is now minimized through the seasonal use of anthelmintics and pasture management, the problem persists in the vast majority of tropical and subtropical regions. Among the gastrointestinal parasites,* Haemonchus contortus* is the species with greatest pathogenic and economic importance in sheep. It is important to assess the type and level of parasitism in ruminant livestock, in order to be able to determine the significance of parasite infection and to recommend the most beneficial and economically acceptable control measures. The determination of the risk factors associated with parasite occurrence can be used to design an effective control strategy [16, 17]. Previously there was not any documented data with regard to the prevalence of the parasite in the study area but there are high populations of sheep in the study area. Therefore, the study was designed with the objective of assessing the prevalence of ovine haemonchosis and its associated risk factors.

## 2. Methods

### 2.1. Study Area

This study was conducted in Wukro which is located 848 km to the North of Addis Ababa and 45 km far from Mekelle town in the eastern administrative zone of Tigray region with an altitude of 13°47′30′′N and longitude of 39°35′57′′E. It is situated in area having an elevation of 1977 m above sea level. The area has clearly defined rainy season from July to September followed by long dry season from October to June. The mean annual rainfall is 610 mm. The minimum and maximum temperature range from 8.3°C to 31°C, respectively [18].

### 2.2. Study Animals

The study animals were 384 indigenous breed of sheep reared using traditional way slaughtered at various restaurants of Wukro Woreda. In this study the origins of the animals were recorded from the history of the animal owners. In addition, the age of the sheep was characterized using teeth eruption Appendix A [19] and body condition scoring method (medium and good) as per [20] Appendix B.

### 2.3. Sample Size Determination

For this study, the total population of sheep in the study area and expected prevalence of 50% was used using the 95% level of confidence. Hence based on the formula indicated by [21] 384 sheep were examined:

(1) $$\begin{array}{c} N=\frac{{\left(1.96\right)}^{2}P\;\;\text{expected}\left(1-P\;\;\text{expected}\right)}{d^{2}}\\ 95\%=\text{level}\;\;\text{of}\;\;\text{confidence}, \end{array}$$

where *n* is sample size (384 animals), *P* is expected prevalence (50%), 1.96 is the value of *Z* of 95% confidence level, and *d* is desired absolute precision = 5%.

### 2.4. Study Design

A cross-sectional study using random sampling was conducted from November 2013 to April 2014 to determine the prevalence of* Haemonchus* species in sheep at the study site.

### 2.5. Data Collection

#### 2.5.1. Antemortem Inspection

Antemortem investigation was performed a few hours before slaughter. The origin, age, sex, body condition, and general health condition of the animal was properly recorded.

#### 2.5.2. Identification of the Worm

The abomasa were removed from their abdominal cavity and ligated the end opening. Then the abomasa were opened along their greater curvature and close visualization was made for the presence of adult* Haemonchus* parasite. The worms were collected in normal saline. Then the parasites were identified based on the characteristics given by [11], Appendix C.

### 2.6. Data Analysis

The data collected were entered to Microsoft Excel spread sheet, coded appropriately, and transferred in to SPSS version 20 software. For data analysis descriptive statistics was employed and statistical significance was considered when *P* value is less than 0.05.

## 3. Results

In this study a total of 384 sheep were examined using postmortem for the presence or absence of* Haemonchus contortus* and the result revealed that 157 (40.9%) were positive.

Similarly, the prevalence of the parasite in sheep having different body condition indicated that there is significance difference of the parasite higher in medium body condition animals with the rate of 27.3% compared to good body condition sheep having the rate of 13.5% (Table 1).

Moreover, there was statistical significant variation in prevalence of* Haemonchus contortus* among the studied age group with higher prevalence in young animals compared to adults in *P* < 0.05 (Table 2).

At the same time there was statistically significant variation in prevalence of* Haemonchus* in male and female with higher rate in male sheep compared to female (*P* < 0.05) (Table 3).

The prevalence of the parasite in sheep that originated from different sites indicates that it was higher in those sheep originated from Negash (25.3%) while the lowest was recorded in sheep originated from Wukro (7.6%) but there was no significant variation (*P* > 0.05) as indicated in Table 4.

## 4. Discussion

The present study revealed that the overall prevalence of* Haemonchus contortus* in sheep was 40.9%, which indicated high prevalence of the parasite in the study area. This finding was much lower compared with the study conducted by [22] in Gonder who reported prevalence of 80.21%. Similarly, the current finding is lowered compared to different researchers such as [23] in Wellega who recorded prevalence of 88.2%, [24] with the rate of 93.6% in the Ogaden region, and [25] 96.5% in Eastern part of Ethiopia. Compared to the finding of [26] who reported the prevalence of 37.18% in Multan abattoir, the current finding was higher.

With regard to the body condition of the examined sheep the rate was higher in medium body condition sheep compared to the good body condition sheep with the prevalence of 27.3% and 13.5%, respectively. This result is inconsistent with the previous reports of [27] who reported prevalence of 77.21% and 84.44% in good and medium body condition animal, respectively. Similarly, [22] indicated that the rate of the parasite was higher in medium body condition sheep compared to that of good body condition with the prevalence of 81.2% and 73.6%, respectively. The prevalence variation of the parasite in these two body conditions varies significantly (*P* < 0.05).

The current finding also indicated that there was significant difference of the prevalence of the parasite in young and adult sheep (*P* < 0.05) where it was higher in young sheep compared to adult sheep with the rates of 21.9% and 19%, respectively, but [27] indicated that the prevalence in young and adult sheep was 86.9% and 86.57%, respectively.

Regarding the sex distribution of the parasite, the current finding revealed that the prevalence in male and female sheep was 29.7% and 11.2%, respectively. There was statistical significant difference in male and female (*P* < 0.05). At the same time, [22] reported that the rate was higher in male with the prevalence of 80.9% compared to female having the rate of 77%. In contrary to the current finding, [28] reported that the rate of the parasite was higher in male compared to female with the prevalence of 34.11% and 39.22%, respectively. In support of the finding of [28] indicated that the rate of the parasite was higher in female than male with the prevalence of 81.9% and 76.9% respectively.

The prevalence of the parasites in sheep that originated from different sites of the study area also indicated that it was higher in those sheep originated from Negash with the rate of 25.3% followed by Agulae and Wukro with the rates of 8.1% and 7.6%, respectively. However there is no statistical difference of the prevalence of the parasite (*P* > 0.05).

## 5. Conclusion and Recommendations

The presence of high rate of* Haemonchus* parasites in the study areas was responsible for the loss of production in sheep. The distribution of the parasites is more common in medium body condition and young animal, which needs great attention when designing the control programs of the parasite. Similarly, the finding showed that most of the male sheep are highly affected. This might be due to the practice of the society and ethical point of slaughtering of the male animals, as most of the female animals are kept for production purposes. Generally, the occurrence of the parasite among the sheep of the study site might be associated with the level of immunity of the animals as most of the young animals and those medium body conditions having low immune status are affected. In addition, the production level of the animals might contribute to the occurrence of the parasite as most of the sheep were using extensive grazing. Based on the current finding, the following points were recommended.

- The strategic deworming should focus on young and poor and medium body condition sheep.
- Improvement of husbandry practices is very important.
- Continuous surveillance of the parasite in sheep farm is essential.
- Further study on the possible risk factors should be conducted.

## Figures and Tables

**Table 1:** Prevalence of *Haemonchus* in sheep based on body condition.

Body condition	Number of animals	Number of positive	*P* value
Medium	175	105 (27.3%)	0.000
Good	209	52 (13.5%)	

Total	384	157 (40.9%)	

**Table 2:** Prevalence of *Haemonchus* in sheep based on the age group.

Age	Number of animals	Number of positive	*P* value
Adult	206	73 (19.0%)	0.019
Young	178	84 (21.9%)	

Total	384	157 (40.9%)	

**Table 3:** Prevalence of *Haemonchus* in sheep based on the sex.

Sex	Number of animals	Number of positive	*P* value
Female	83	43 (11.2%)	0.022
Male	301	114 (29.7%)	

Total	384	157 (40.9%)	

**Table 4:** Prevalence of* Haemonchus* in sheep based on the origin.

Origin	Number of animals	Number of positive	*P* value
Negash	210	97 (25.3%)	0.066
Wukro	86	29 (7.6%)	
Agulae	88	31 (8.1%)	

Total	384	157 (40.9%)	

**Table 5:** 

Age	Permanent incisors
Less than 1 year (young)	0
Greater than 1 year (adult)	1 and greater than 1 pair

Source: [19].

## Appendices

### A. Age: Based on Teeth Eruption

See Table 5.

### B. Body Condition Score

*Body Condition Score*. It is sased on visual assessment on postmortem in which scoring is based on the level of fat deposition and muscle around over the vertebrate in the loin region and omental fat deposition [20].

*Poor*. Poor was characterized as no or little fat cover, no full muscle, sbsence or little omental fat prominent spinous, and transversal process which are from distant.

*Medium*. Medium was characterized as moderate fat cover, full muscle, and moderate omental fat, spinous and transverse process are detected up on palpation.

*Good*. Good was characterized as thick or very fat cover, full muscle, and thick omental fat, and transverse process cannot be detected and spinous process detected as a hard line.

### C. *Haemonchus* Identification

*Gross Characteristics*. The adult are easily identified because of their specific location in the abomasa, their large size (2-3 centimeters), and their color (reddish in color). In fresh specimen, the white ovaries winding spirally around the blood filled intestine produce a “barbed pole” appearance [11].
